# Editorial for Special Issue “Heavy Metals Accumulation, Toxicity, and Detoxification in Plants” †

**DOI:** 10.3390/ijms21114103

**Published:** 2020-06-09

**Authors:** Alessio Aprile, Luigi De Bellis

**Affiliations:** Department of Biological and Environmental Sciences and Technologies, University of Salento, I-73100 Lecce, Italy; alessio.aprile@unisalento.it

“Heavy metals” is a collective term widely applied for the group of metals and metalloids with an atomic density above 4 g/cm^3^ [1]. Non-essential toxic plant heavy metals include arsenic (As), cadmium (Cd), chromium (Cr), cobalt (Co), lead (Pb), mercury (Hg), nickel (Ni), and vanadium (V); whereas others are essential, such as copper (Cu), iron (Fe), manganese (Mn), and zinc (Zn). Heavy metals cause harmful effects in plants, animals, and humans as a result of long-term or acute exposure. Toxicity from heavy metals is increasing due to the extensive release from industrial, agricultural, chemical, domestic, and technological sources, which in turn contaminate the water, soil, and air. Natural phenomena, such as volcanic eruptions and sea movements, also contribute to the natural cyclization of metals on the earth, and human activities often alter the rate of release and transport by increasing emissions by a few orders of magnitude. 

Heavy metals penetrate the human body through water, food, and air. Inside an organism, they bind to cellular structures, thereby damaging the performance of essential biological functions. Metals, for example, easily bind to the sulfhydryl groups of several enzymes that control the speed of metabolic reactions: the “new” metal-enzyme complex leads to the loss of the catalytic activity of the enzyme. The level of toxicity from heavy metals depends on several factors, including time of exposure, dose, and the health status of the people exposed.

The European Environment Agency (EEA) reported that of the 1000 industrial plants that released heavy metals into the air in 2016, eighteen accounted for more than half of the total pollution, suggesting a great responsibility on the part of a few large companies (Figure 1) [2]. 

An additional issue is the biomagnification (or bioaccumulation) caused by the very slow rate of elimination of heavy metals from an organism. Bioaccumulation, in ecology and biology, is the process whereby the accumulation of toxic substances in living beings increases in concentration following a rise in the trophic level: the higher the trophic level, the stronger the concentration of heavy metals. Biomagnification is also expressed as the concentration increase of a pollutant in a biological organism over time. 

To limit the risks for humans and the environment, many countries have legislated limits for each heavy metal. Specific limits have been defined in drinking, waste, and surface waters (lakes, rivers, seas). There are also limits in foods and animal feed, because heavy metals can easily enter the food chain through plants (or algae) and are subsequently bioaccumulated into the higher trophic levels. The risk for human health is due to directly eating edible plant tissues, or indirectly through eating animals that have in turn fed on herbivores or directly on edible plant tissues. Understanding the mechanisms for regulating the storage and distribution of heavy metals in plants is the basis for improving the safety of the food chain.

This special issue, entitled “Heavy Metals Accumulation, Toxicity, and Detoxification in Plants”, explores three main issues concerning heavy metals: (a) the accumulation and partitioning of heavy metals in crops and wild plants; (b) the toxicity and molecular behaviors of cells, tissues, and their effects on physiology and plant growth; and (c) detoxification strategies, plant tolerance, and phytoremediation. 

The issue contains a total of 19 articles (Table 1). There are four reviews covering the following topics: phytoremediation [3], manganese phytotoxicity in plants [4], cadmium effect on plant development [5], the genetic characteristics of Cd accumulation and the research status of genes and quantitative trait loci (QTLs) in rice [6], and fifteen original research articles, mainly regarding the impact of cadmium on plants [7,8,9,10,11,12,13,14,15,16,17,18,19,20,21].

Cadmium is therefore the predominant topic of this special issue, thus confirming the focus of the research community on the negative impacts determined by cadmium or cadmium associated with other heavy metals. Interestingly, we did not receive any manuscripts on other heavy metals such as arsenic, chromium and mercury despite their danger for human health. 

The cadmium research articles come from China, Poland, Italy, Canada, Pakistan, and the United States. These studies investigate different molecular mechanisms or approaches, using model plants such as *Arabidopsis* and tobacco [17,18,20] or hyperaccumulator plant species [9,16,19,21] to unravel their molecular strategies in heavy metal accumulation. Other articles focus on how to prevent cadmium from entering the food chain by investigating edible plants such as *Zea mays* [7], durum and bread wheat [12,13], or animal feeding plants such as *Lolium multiflorum*.

The studies reveal some common strategies in terms of the molecular mechanisms involved. Some plants activate the production of small proteins such as glutathione S-transferase (GST) and small heat shock protein (sHSP) [9,11,21] or antioxidants [16]. In order to alleviate heavy metal toxicity, other plants respond by activating a complex metabolism-like auxin pathway [7,8,17]. Plants also produce specific metallothionines and phytosiderophores [10,12] to chelate heavy metals or to activate heavy metals transporters such as heavy metal ATPase (e.g., HMA2 and HMA4) and ATP-binding cassette (ABC) transporters [12,13,18,19,21].

The studies in this special issue highlight considerable genetic variability, suggesting different possibilities for accumulation, translocation, and reducing or controlling heavy metals toxicity in plants.

Heavy metal pollution is still one of the world’s great challenges. In the future, the main research objective should be to identify and characterize the genes controlling the uptake and translocation of heavy metals in a plant’s above-ground organs in order to produce (i) phytoremediation plants that efficiently move heavy metals in the stem and leaves or (ii) plants dedicated to human nutrition that transport heavy metals only in trace amounts to seeds or fruits.

## Figures and Tables

**Figure 1:**
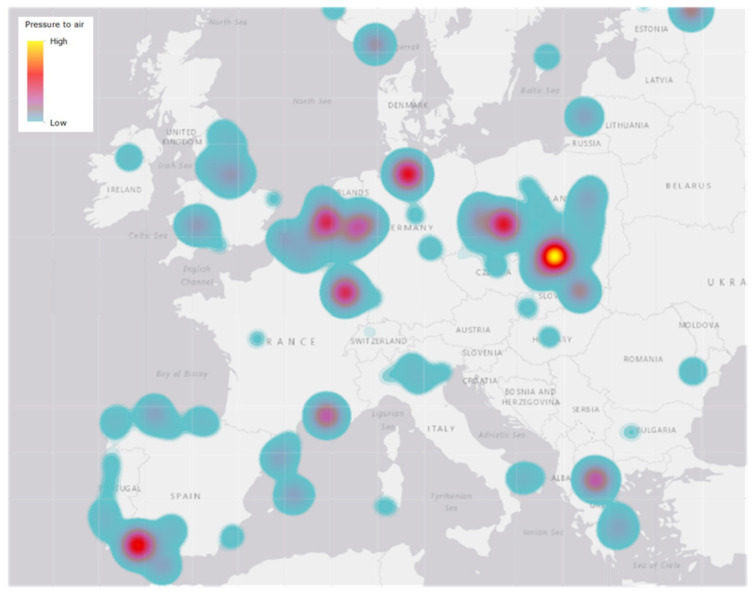
Environmental pressures of heavy metal releases to air, 2016 [2]. An eco-toxicity approach (USEtox model, https://usetox.org/model) was applied to illustrate spatially the combined environmental pressures on Europe’s environment caused by releases of the selected pollutants. This gives information about the location of source of heavy metals and the low or high levels in air as indicated in the upper left corner of the figure.

**Table 1:** Contributors to the special issue “Heavy Metals Accumulation, Toxicity, and Detoxification in Plants”. ABC: ATP-binding cassette.

Authors	Title	Heavy Metals	Type
Małkowski et al. [7]	Hormesis in Plants: The Role of Oxidative Stress, Auxins and Photosynthesis in Corn Treated with Cd or Pb	Cadmium Lead	Original Research
Hu et al. [8]	Full-Length Transcriptome Assembly of Italian Ryegrass Root Integrated with RNA-Seq to Identify Genes in Response to Plant Cadmium Stress	Cadmium	Original Research
Sun et al. [9]	Comparative Transcriptome Analysis of the Molecular Mechanism of the Hairy Roots of *Brassica campestris* L. in Response to Cadmium Stress	Cadmium	Original Research
Zúñiga et al. [10]	Isolation and Characterization of Copper- and Zinc-Binding Metallothioneins from the Marine Alga *Ulva compressa* (Chlorophyta)	Copper, Zinc	Original Research
Cui et al. [11]	OsMSR3, a Small Heat Shock Protein, Confers Enhanced Tolerance to Copper Stress in *Arabidopsis thaliana*	Copper	Original Research
Aprile et al. [12]	Combined Effect of Cadmium and Lead on Durum Wheat	Cadmium, Lead	Original Research
Shafiq et al. [13]	Lead, Cadmium and Zinc Phytotoxicity Alter DNA Methylation Levels to Confer Heavy Metal Tolerance in Wheat	Cadmium, Lead, Zinc	Original Research
Celis-Plá et al. [14]	MAPK Pathway under Chronic Copper Excess in Green Macroalgae (Chlorophyta): Influence on Metal Exclusion/Extrusion Mechanisms and Photosynthesis	Copper	Original Research
Rodríguez-Rojas et al. [15]	MAPK Pathway under Chronic Copper Excess in Green Macroalgae (Chlorophyta): Involvement in the Regulation of Detoxification Mechanisms	Copper	Original Research
Małecka et al. [16]	Insight into the Phytoremediation Capability of *Brassica juncea* (v. Malopolska): Metal Accumulation and Antioxidant Enzyme Activity	Cadmium, Copper, Lead, Zinc	Original Research
Luo et al. [17]	Selenium Modulates the Level of Auxin to Alleviate the Toxicity of Cadmium in Tobacco	Cadmium	Original Research
Wang et al. [18]	Ectopic Expression of Poplar ABC Transporter PtoABCG36 Confers Cd Tolerance in *Arabidopsis thaliana*	Cadmium	Original Research
Shu et al. [19]	Comparative Transcriptomic Studies on a Cadmium Hyperaccumulator *Viola baoshanensis* and Its Non-Tolerant Counterpart *V. inconspicua*	Cadmium	Original Research
He et al. [20]	Exogenous Glycinebetaine Reduces Cadmium Uptake and Mitigates Cadmium Toxicity in Two Tobacco Genotypes Differing in Cadmium Tolerance	Cadmium	Original Research
Han et al. [21]	Transcriptome Analysis Reveals Cotton (*Gossypium hirsutum*) Genes That Are Differentially Expressed in Cadmium Stress Tolerance	Cadmium	Original Research
Li et al. [4]	Advances in the Mechanisms of Plant Tolerance to Manganese Toxicity	Manganese	Review
Huybrechts et al. [5]	Cadmium and Plant Development: An Agony from Seed to Seed	Cadmium	Review
Chen et al. [6]	Advances in the Uptake and Transport Mechanisms and QTLs Mapping of Cadmium in Rice	Cadmium	Review
Dal Corso et al. [3]	Heavy Metal Pollutions: State of the Art and Innovation in Phytoremediation	All	Review

## Abbreviations

QTLs	Quantitative trait loci
sHSP	small heat shock protein
GST	glutathione s-transferase
HMA	heavy metal ATPase
ABC	ATP-binding cassette
